# Development of a machine learning tool to predict the risk of incident chronic kidney disease using health examination data

**DOI:** 10.3389/fpubh.2024.1495054

**Published:** 2024-11-01

**Authors:** Yuki Yoshizaki, Kiminori Kato, Kazuya Fujihara, Hirohito Sone, Kohei Akazawa

**Affiliations:** ^1^Department of Medical Informatics and Statistics, Niigata University Graduate School of Medical and Dental Sciences, Niigata, Japan; ^2^Department of Prevention of Noncommunicable Diseases and Promotion of Health Checkup, Niigata University Graduate School of Medical and Dental Sciences, Niigata, Japan; ^3^Department of Hematology, Endocrinology and Metabolism, Niigata University Graduate School of Medical and Dental Sciences, Niigata, Japan; ^4^Department of Medical Informatics, Niigata University Medical and Dental Hospital, Niigata, Japan

**Keywords:** chronic kidney disease, health examination, estimated glomerular filtration rate, proteinuria, recurrent neural network

## Abstract

**Background:**

Chronic kidney disease (CKD) is characterized by a decreased glomerular filtration rate or renal injury (especially proteinuria) for at least 3 months. The early detection and treatment of CKD, a major global public health concern, before the onset of symptoms is important. This study aimed to develop machine learning models to predict the risk of developing CKD within 1 and 5 years using health examination data.

**Methods:**

Data were collected from patients who underwent annual health examinations between 2017 and 2022. Among the 30,273 participants included in the study, 1,372 had CKD. Demographic characteristics, body mass index, blood pressure, blood and urine test results, and questionnaire responses were used to predict the risk of CKD development at 1 and 5 years. This study examined three outcomes: incident estimated glomerular filtration rate (eGFR) <60 mL/min/1.73 m^2^, the development of proteinuria, and incident eGFR <60 mL/min/1.73 m^2^ or the development of proteinuria. Logistic regression (LR), conditional logistic regression, neural network, and recurrent neural network were used to develop the prediction models.

**Results:**

All models had predictive values, sensitivities, and specificities >0.8 for predicting the onset of CKD in 1 year when the outcome was eGFR <60 mL/min/1.73 m^2^. The area under the receiver operating characteristic curve (AUROC) was >0.9. With LR and a neural network, the specificities were 0.749 and 0.739 and AUROCs were 0.889 and 0.890, respectively, for predicting onset within 5 years. The AUROCs of most models were approximately 0.65 when the outcome was eGFR <60 mL/min/1.73 m^2^ or proteinuria. The predictive performance of all models exhibited a significant decrease when eGFR was not included as an explanatory variable (AUROCs: 0.498–0.732).

**Conclusion:**

Machine learning models can predict the risk of CKD, and eGFR plays a crucial role in predicting the onset of CKD. However, it is difficult to predict the onset of proteinuria based solely on health examination data. Further studies must be conducted to predict the decline in eGFR and increase in urine protein levels.

## 1 Introduction

Chronic kidney disease (CKD) is characterized by a decreased glomerular filtration rate or renal injury (especially proteinuria) for at least 3 months and is a significant global public health concern. The number of patients with CKD worldwide reached 697.5 million in 2017, yielding a prevalence rate of 9.1% (1). The number of patients with CKD has been increasing in Japan. Approximately 14.8 million individuals in Japan had CKD in 2015, with a prevalence rate of 14.6% (2).

Symptoms such as edema and abnormal urine are usually not observed during the early stages of CKD. Notably, irreversible decline in renal function has already occurred in most patients by the time they experience symptoms. Dialysis is indicated in patients with a significant decline in renal function. Dialysis leads to a decline in quality of life. Furthermore, it creates social burden by increasing medical expenses. Thus, early detection and treatment of CKD before symptom onset are important.

Hypertension, diabetes, dyslipidemia, hyperuricemia, metabolic syndrome, and obesity are known risk factors for CKD (3–7) that are strongly associated with undesirable lifestyle habits, such as lack of exercise and physical activity, inappropriate eating habits, smoking, excessive drinking, and inadequate sleep (8–13). Many of these lifestyle factors affect the risk of developing CKD (3, 14–17).

Previous studies explored the development of CKD prediction models using machine learning. Shih et al. (18) predicted the risk of early CKD using a classification and regression tree (area under the receiver operating characteristics curve [AUROC]: 0.779), C4.5 decision tree (decision tree algorithm; AUROC: 0.788), linear discriminant analysis (AUROC: 0.773), and neural network (NN) (AUROC: 0.692). A lifestyle scoring system for identifying CKD risk was established using a light-gradient boosting machine algorithm in a previous study that investigated the relationship between CKD and lifestyle habits (19). The AUROC for predicting the incidence of CKD based on lifestyle scores was 0.710. Recurrent neural network (RNN), which is suitable for analyzing time-series data, was used to predict disease progression among patients with CKD in another study (20).

This study aimed to develop machine learning models to predict the risk of CKD onset within 1 and 5 years using health examination data. Many previous studies that predicted the risk of CKD attempted to predict only the risk of a decline in eGFR, which is not considered a strict predictor of the risk of CKD. Thus, three outcomes were analyzed in this study: decline in eGFR, presence of renal injury, and decline in eGFR or presence of renal injury. These outcomes were compared to determine their contribution to predicting renal function decline and renal injury toward CKD screening. Lifestyle questionnaire data were used along with demographic characteristics, physical measurements, and laboratory data to predict the risk of CKD. Individuals are expected to implement ideal lifestyle habits and avoid undesirable lifestyle habits when the risk of developing CKD is predicted using these variables. Finally, annual health examination data were used to determine the importance of undergoing annual health examinations.

## 2 Methods

### 2.1 Study participants

The Niigata Association of Occupational Health has multiple health examination centers in Niigata Prefecture, Japan, and provides routine health and complete medical examinations. The health examination data of individuals who underwent annual health examinations at the Niigata Association of Occupational Health between 2017 and 2022 were used in this study. Data that met the following criteria were extracted from the Niigata Association of Occupational Health database.

**Figure 1:**
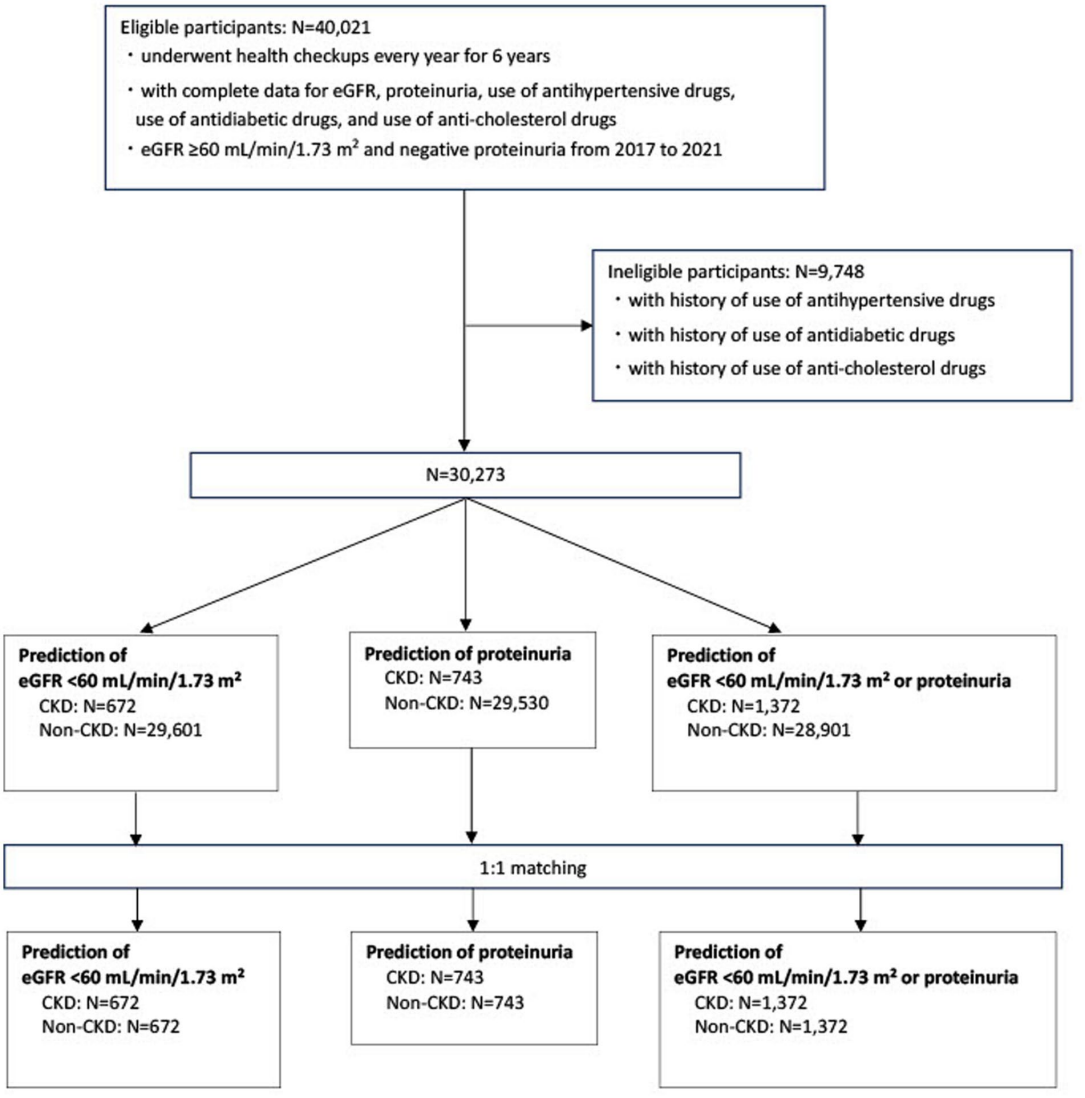
Flow diagram for the selection of study participants eGFR, estimated glomerular filtration rate; CKD, chronic kidney disease.

Figure 1 illustrates the participant selection process. Participants who underwent health examinations every year for 6 years and had an eGFR of ≥60 mL/min/1.73 m^2^ and negative proteinuria from 2017 to 2021 were included in the study (*N* = 40,021). They had complete data on eGFR, proteinuria, and the use of antihypertensive, antidiabetic, and anti-cholesterol drugs. Participants with a history of antihypertensive, antidiabetic, or anticholesterol drug use were excluded (*N* = 9,748). Therefore, 30,273 participants were included in the final analysis. Risk prediction models were created for the following outcomes (21): (i) incident eGFR of <60 mL/min/1.73 m^2^ in 2022, (ii) development of proteinuria in 2022, and (iii) incident eGFR of <60 mL/min/1.73 m^2^ or development of proteinuria in 2022.

In addition, participants with and without CKD were matched according to age and sex in a 1:1 ratio. Each patient with CKD was matched with an age-and sex-matched participant without CKD, selected randomly and non-restoratively.

### 2.2 Data collection

Data regarding the following parameters were collected: (i) demographic characteristics, including age and sex; (ii) clinical parameters, such as body mass index (kg/m^2^), systolic blood pressure (mmHg), and diastolic blood pressure (mmHg); (iii) biochemical indicators, including fasting plasma glucose levels (mg/dL), hemoglobin A1c levels (%), total cholesterol levels (mg/dL), triglyceride levels (mg/dL), high-density lipoprotein cholesterol levels (mg/dL), low-density lipoprotein cholesterol levels (mg/dL), uric acid levels (mg/dL), serum creatinine levels (mg/dL), eGFR (mL/min/1.73 m^2^; calculated using a formula for Japanese individuals (22)), and proteinuria (dipstick urinalysis); (iv) information obtained from a questionnaire, such as the use of antihypertensive drugs (yes or no), the use of antidiabetic drugs (yes or no), the use of anti-cholesterol drugs (yes or no), smoking status, exercise (yes or no), physical activity (yes or no), late dinner (eating dinner <2 h before bedtime) (yes or no), snacking (yes or no), skipping breakfast (yes or no), adequate sleep, i.e., getting enough rest through sleep (yes or no), eating speed, i.e., eating faster than others (fast or normal or slow), drinking frequency (seldom [cannot drink], sometimes, or every day), and alcohol intake (<20 g/day, 20–40 g/day, 40–60 g/day, or ≥ 60 g/day).

### 2.3 Statistical analysis and machine learning

Missing data were imputed using a multiple imputation method based on random forest (23).

The machine learning methods, RNN and NN, and the traditional statistical methods logistic regression (LR) and conditional logistic regression (CLR) were used to construct the prediction models.

The NN and LR models were used for predictions based on unmatched data for 2017 and 2021. The RNN, NN, and LR models were used for predictions based on unmatched 5-year data from 2017 to 2021. The LR model was substituted with the CLR model when the matched data were analyzed.

The outcome variables included incident eGFR <60 mL/min/1.73 m^2^ in 2022, proteinuria in 2022, and incident eGFR <60 mL/min/1.73 m^2^ or proteinuria in 2022. The candidate variables, except proteinuria, were treated as explanatory variables.

The training and testing samples used in this study comprised 80 and 20% of the entire analyzed dataset, respectively. The prediction models were developed using training samples. The accuracy, sensitivity, specificity, and AUROC of the testing sample were used to evaluate the performance of the model.

The NN models were trained using three hidden layers with 256 nodes in each hidden layer. The RNN models were trained using four hidden layers with 16 nodes in each hidden layer.

Tensorflow 2.15.0 and scikit-learn 1.3.2 (24) were used to implement the procedures for the development of the models.

## 3 Results

Tables 1–3 present the baseline characteristics (data from 2017) of the participants. Continuous variables are presented as mean ± standard deviation, whereas categorical variables are presented as frequencies and proportions. All participants had a dipstick urinalysis score of (−); consequently, proteinuria was not included as an explanatory variable.

**Table 1:** Baseline characteristics in the prediction of eGFR <60 mL/min/1.73 m^2^.

	Before matching		After matching	
Variables	CKD (*n* = 672)	Non-CKD (*n* = 29,601)	CKD (*n* = 672)	Non-CKD (*n* = 672)
Age, years	50.1 ± 8.1	44.5 ± 9.4	50.1 ± 8.1	50.1 ± 8.1
Male, *n* (%)	396 (58.9)	18,748 (63.3)	396 (58.9)	396 (58.9)
eGFR, mL/min/m^2^	71.3 ± 6.6	88.4 ± 14.1	71.3 ± 6.6	85.6 ± 13.4
Body mass index, kg/m^2^	22.7 ± 3.3	22.2 ± 3.3	22.7 ± 3.3	22.1 ± 3.1
Systolic blood pressure, mmHg	121.6 ± 14.3	119.4 ± 13.4	121.6 ± 14.3	120.0 ± 14.4
Diastolic blood pressure, mmHg	76.7 ± 10.4	74.3 ± 10.2	76.7 ± 10.4	75.4 ± 10.2
Fasting plasma glucose, mg/dL	92.2 ± 8.5	91.0 ± 8.4	92.2 ± 8.5	92.7 ± 9.0
Hemoglobin A1c, %	5.5 ± 0.2	5.5 ± 0.2	5.5 ± 0.2	5.5 ± 0.2
Total cholesterol, mg/dL	211.9 ± 32.2	204.9 ± 33.3	211.9 ± 32.2	211.0 ± 31.9
Triglycerides, mg/dL	110.2 ± 89.3	98.4 ± 77.4	110.2 ± 89.3	96.4 ± 64.7
HDL-C, mg/dL	64.6 ± 16.5	64.9 ± 16.1	64.6 ± 16.5	66.0 ± 15.7
LDL-C, mg/dL	124.4 ± 30.5	119.8 ± 29.8	124.4 ± 30.5	124.3 ± 28.4
Uric acid, mg/dL	5.6 ± 1.4	5.4 ± 1.4	5.6 ± 1.4	5.2 ± 1.3
Smoking, *n* (%)				
Yes	200 (29.8)	10,319 (34.9)	200 (29.8)	225 (33.5)
Exercise, *n* (%)				
Yes	133 (19.8)	5,012 (16.9)	133 (19.8)	106 (15.8)
Physical activity, *n* (%)				
Yes	218 (32.4)	10,222 (34.5)	218 (32.4)	232 (34.5)
Eating speed, *n* (%)				
Slow	188 (28.0)	8,311 (28.1)	188 (28.0)	174 (25.9)
Normal	426 (63.4)	18,594 (62.8)	426 (63.4)	438 (65.2)
Fast	58 (8.6)	2,696 (9.1)	58 (8.6)	60 (8.9)
Late dinner, *n* (%)				
Yes	180 (26.8)	9,153 (30.9)	180 (26.8)	190 (28.3)
Snacking, *n* (%)				
Yes	126 (18.8)	5,594 (18.9)	126 (18.8)	130 (19.3)
Skipping breakfast, *n* (%)				
Yes	107 (15.9)	6,631 (22.4)	107 (15.9)	140 (20.8)
Drinking frequency, *n* (%)				
Seldom (cannot drink)	265 (39.4)	10,799 (36.5)	265 (39.4)	251 (37.4)
Sometimes	216 (32.1)	9,277 (31.3)	216 (32.1)	202 (30.1)
Every day	191 (28.4)	9,525 (32.2)	191 (28.4)	219 (32.6)
Alcohol intake, *n* (%)				
<20 g/day	410 (61.0)	16,969 (57.3)	410 (61.0)	390 (58.0)
20–40 g/day	197 (29.3)	8,641 (29.2)	197 (29.3)	191 (28.4)
40–60 g/day	51 (7.6)	3,126 (10.6)	51 (7.6)	74 (11.0)
≥60 g/day	14 (2.1)	865 (2.9)	14 (2.1)	17 (2.5)
Adequate sleep, *n* (%)				
Yes	439 (65.3)	18,855 (63.7)	439 (65.3)	437 (65.0)

Continuous variables are expressed as mean ± standard deviation. Categorical variables are expressed as frequencies and proportions.

CKD, chronic kidney disease; eGFR, estimated glomerular filtration rate; HDL-C, high-density lipoprotein cholesterol; LDL-C, low-density lipoprotein cholesterol.

**Table 2:** Baseline characteristics in the prediction of proteinuria.

	Before matching		After matching	
Variables	CKD (*n* = 743)	Non-CKD (*n* = 29,530)	CKD (*n* = 743)	Non-CKD (*n* = 743)
Age, years	44.3 ± 9.1	44.7 ± 9.4	44.3 ± 9.1	44.3 ± 9.1
Male, n (%)	488 (65.7)	18,656 (63.2)	488 (65.7)	488 (65.7)
eGFR, mL/min/m^2^	88.0 ± 13.6	88.0 ± 14.2	88.0 ± 13.6	88.4 ± 13.7
Body mass index, kg/m^2^	22.9 ± 3.6	22.2 ± 3.3	22.9 ± 3.6	22.2 ± 3.4
Systolic blood pressure, mmHg	121.1 ± 14.0	119.4 ± 13.4	121.1 ± 14.0	119.6 ± 14.0
Diastolic blood pressure, mmHg	75.5 ± 10.8	74.4 ± 10.2	75.5 ± 10.8	74.6 ± 10.0
Fasting plasma glucose, mg/dL	92.1 ± 9.5	91.0 ± 8.4	92.1 ± 9.5	91.3 ± 10.3
Hemoglobin A1c, %	5.5 ± 0.2	5.5 ± 0.2	5.5 ± 0.2	5.5 ± 0.2
Total cholesterol, mg/dL	205.0 ± 33.7	205.1 ± 33.3	205.0 ± 33.7	203.7 ± 33.0
Triglycerides, mg/dL	106.5 ± 78.2	98.5 ± 77.7	106.5 ± 78.2	105.5 ± 92.7
HDL-C, mg/dL	62.9 ± 16.3	65.0 ± 16.1	62.9 ± 16.3	64.1 ± 16.5
LDL-C, mg/dL	121.1 ± 31.3	119.9 ± 29.8	121.1 ± 31.3	118.9 ± 28.8
Uric acid, mg/dL	5.3 ± 1.3	5.4 ± 1.4	5.3 ± 1.3	5.4 ± 1.4
Smoking, *n* (%)				
Yes	287 (38.6)	10,232 (34.6)	287 (38.6)	278 (37.4)
Exercise, *n* (%)				
Yes	122 (16.4)	5,023 (17.0)	122 (16.4)	124 (16.7)
Physical activity, *n* (%)				
Yes	245 (33.0)	10,195 (34.5)	245 (33.0)	257 (34.6)
Eating speed, *n* (%)				
Slow	198 (26.6)	8,301 (28.1)	198 (26.6)	223 (30.0)
Normal	478 (64.3)	18,542 (62.8)	478 (64.3)	455 (61.2)
Fast	67 (9.0)	2,687 (9.1)	67 (9.0)	65 (8.7)
Late dinner, *n* (%)				
Yes	234 (31.5)	9,099 (30.8)	234 (31.5)	241 (32.4)
Snacking, *n* (%)				
Yes	140 (18.8)	5,580 (18.9)	140 (18.8)	140 (18.8)
Skipping breakfast, *n* (%)				
Yes	195 (26.2)	6,543 (22.2)	195 (26.2)	160 (21.5)
Drinking frequency, *n* (%)				
Seldom (cannot drink)	307 (41.3)	10,757 (36.4)	307 (41.3)	227 (37.3)
Sometimes	227 (30.6)	9,266 (31.4)	227 (30.6)	228 (30.7)
Every day	209 (28.1)	9,507 (32.2)	209 (28.1)	238 (32.0)
Alcohol intake, *n* (%)				
<20 g/day	443 (59.6)	16,936 (57.4)	443 (59.6)	447 (60.2)
20–40 g/day	200 (26.9)	8,638 (29.3)	200 (26.9)	193 (26.0)
40–60 g/day	70 (9.4)	3,107 (10.5)	70 (9.4)	83 (11.2)
≥60 g/day	30 (4.0)	849 (2.9)	30 (4.0)	20 (2.7)
Adequate sleep, *n* (%)				
Yes	465 (62.6)	18,829 (63.8)	465 (62.6)	462 (62.2)

Continuous variables are expressed as mean ± standard deviation. Categorical variables are expressed as frequencies and proportions.

CKD, chronic kidney disease; eGFR, estimated glomerular filtration rate; HDL-C, high-density lipoprotein cholesterol; LDL-C, low-density lipoprotein cholesterol.

**Table 3:** Baseline characteristics in the prediction of eGFR <60 mL/min/1.73 m^2^ or proteinuria.

	Before matching		After matching	
Variables	CKD (*n* = 1,372)	Non-CKD (*n* = 28,901)	CKD (*n* = 1,372)	Non-CKD (*n* = 1,372)
Age, years	47.0 ± 9.2	44.5 ± 9.4	47.0 ± 9.2	47.0 ± 9.2
Male, *n* (%)	854 (62.2)	18,290 (63.3)	854 (62.2)	854 (62.2)
eGFR, mL/min/m^2^	80.3 ± 13.8	88.4 ± 14.1	80.3 ± 13.8	86.6 ± 14.1
Body mass index, kg/m^2^	22.8 ± 3.5	22.1 ± 3.3	22.8 ± 3.5	22.2 ± 3.2
Systolic blood pressure, mmHg	121.2 ± 14.1	119.3 ± 13.4	121.2 ± 14.1	119.5 ± 13.3
Diastolic blood pressure, mmHg	75.9 ± 10.6	74.3 ± 10.2	75.9 ± 10.6	75.2 ± 10.3
Fasting plasma glucose, mg/dL	92.1 ± 9.0	91.0 ± 8.4	92.1 ± 9.0	91.7 ± 8.4
Hemoglobin A1c, %	5.5 ± 0.2	5.5 ± 0.2	5.5 ± 0.2	5.5 ± 0.2
Total cholesterol, mg/dL	208.0 ± 33.0	204.9 ± 33.3	208.0 ± 33.0	208.1 ± 33.8
Triglycerides, mg/dL	107.6 ± 83.6	98.3 ± 77.4	107.6 ± 83.6	100.4 ± 76.6
HDL-C, mg/dL	63.7 ± 16.4	65.0 ± 16.1	63.7 ± 16.4	65.4 ± 16.5
LDL-C, mg/dL	122.6 ± 30.8	119.8 ± 29.7	122.6 ± 30.8	121.6 ± 29.7
Uric acid, mg/dL	5.4 ± 1.4	5.4 ± 1.4	5.4 ± 1.4	5.4 ± 1.4
Smoking, *n* (%)				
Yes	467 (34.0)	10,052 (34.8)	467 (34.0)	469 (34.2)
Exercise, *n* (%)				
Yes	250 (18.2)	4,895 (16.9)	250 (18.2)	224 (16.3)
Physical activity, *n* (%)				
Yes	449 (32.7)	9,991 (34.6)	449 (32.7)	432 (31.5)
Eating speed, *n* (%)				
Slow	376 (27.4)	8,123 (28.1)	376 (27.4)	371 (27.0)
Normal	876 (63.8)	18,144 (62.8)	876 (63.8)	872 (63.6)
Fast	120 (8.7)	2,634 (9.1)	120 (8.7)	129 (9.4)
Late dinner, *n* (%)				
Yes	405 (29.5)	8,928 (30.9)	405 (29.5)	404 (29.4)
Snacking, *n* (%)				
Yes	256 (18.7)	5,464 (18.9)	256 (18.7)	265 (19.3)
Skipping breakfast, *n* (%)				
Yes	288 (21.0)	6,450 (22.3)	288 (21.0)	289 (21.1)
Drinking frequency, *n* (%)				
Seldom (cannot drink)	553 (40.3)	10,511 (36.4)	553 (40.3)	478 (34.8)
Sometimes	430 (31.3)	9,063 (31.4)	430 (31.3)	402 (29.3)
Every day	389 (28.4)	9,327 (32.3)	389 (28.4)	492 (35.9)
Alcohol intake, *n* (%)				
<20 g/day	824 (60.1)	16,555 (57.3)	824 (60.1)	777 (56.6)
20–40 g/day	389 (28.4)	8,449 (29.2)	389 (28.4)	403 (29.4)
40–60 g/day	116 (8.5)	3,061 (10.6)	116 (8.5)	153 (11.2)
≥60 g/day	43 (3.1)	836 (2.9)	43 (3.1)	39 (2.8)
Adequate sleep, *n* (%)				
Yes	872 (63.6)	18,422 (63.7)	872 (63.6)	871 (63.5)

Continuous variables are expressed as mean ± standard deviation. Categorical variables are expressed as frequencies and proportions.

CKD, chronic kidney disease; eGFR, estimated glomerular filtration rate; HDL-C, high-density lipoprotein cholesterol; LDL-C, low-density lipoprotein cholesterol.

**Figure 2:**
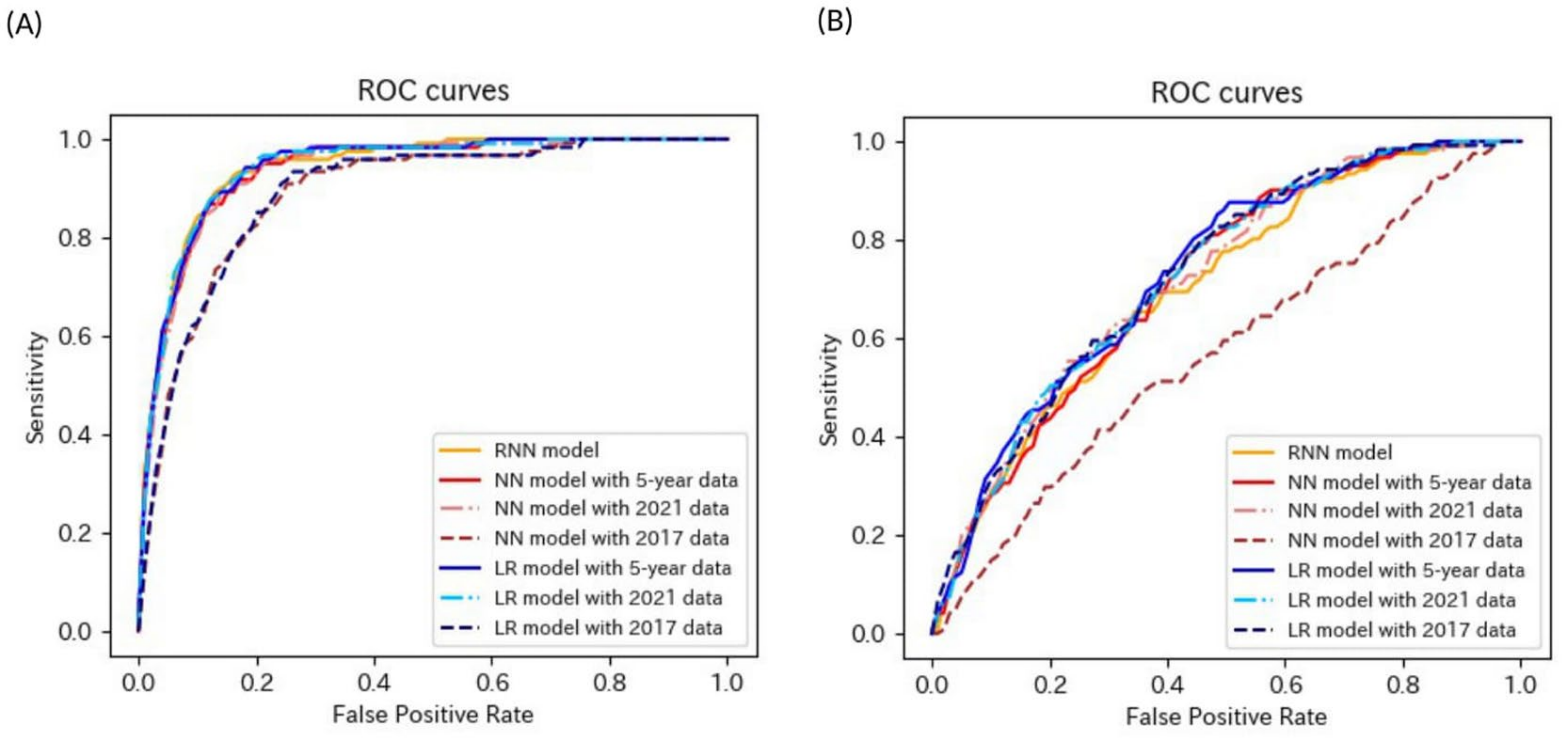
ROC curves of the models for the prediction of eGFR <60 mL/min/1.73 m^2^ in non-matched data analysis. (A) When all candidate variables are used as explanatory variables. (B) When eGFR was excluded from the explanatory variables. ROC, receiver operating characteristics; eGFR, estimated glomerular filtration rate; RNN, recurrent neural network; NN, neural network; LR, logistic regression.

**Figure 3:**
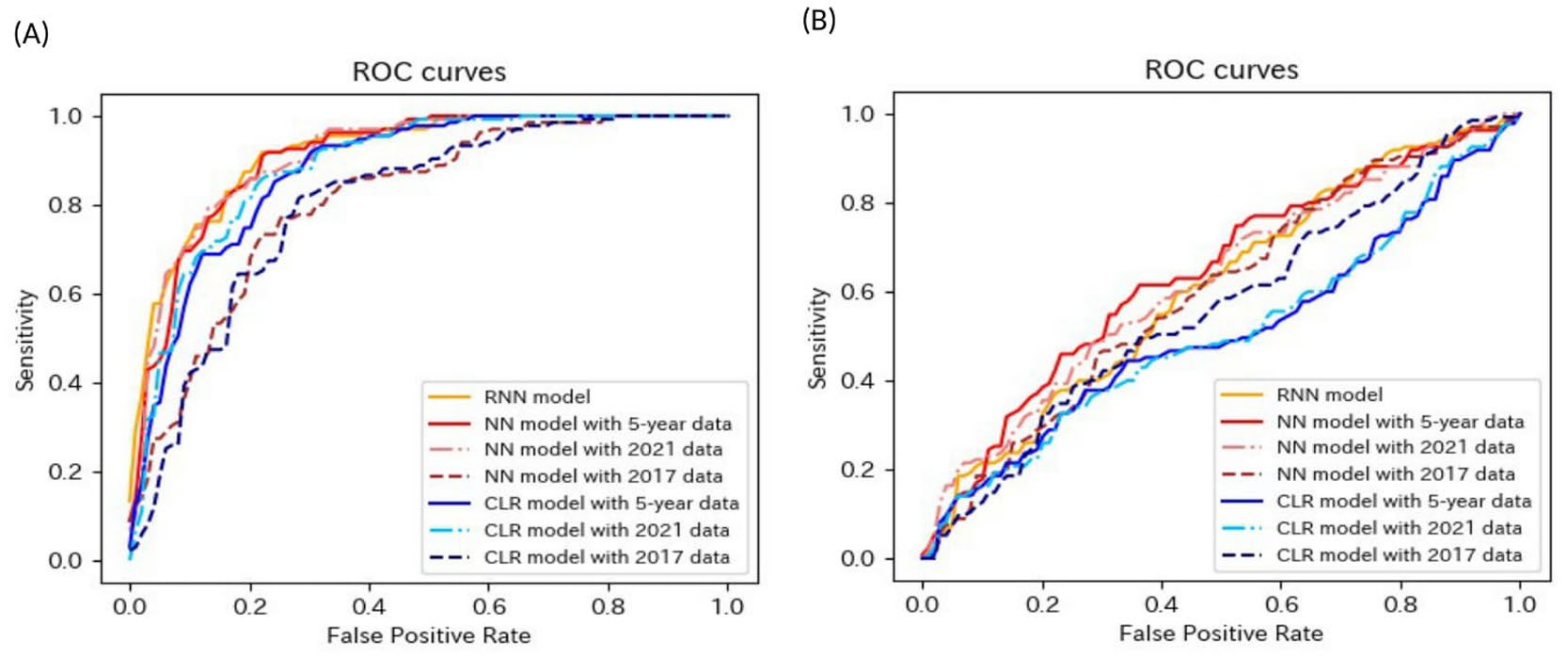
ROC curves of the models for the prediction of eGFR <60 mL/min/1.73 m^2^ in matched data analysis. (A) When all candidate variables are used as explanatory variables. (B) When eGFR was excluded from the explanatory variables. ROC, receiver operating characteristics; eGFR, estimated glomerular filtration rate; RNN, recurrent neural network; NN, neural network; CLR, conditional logistic regression.

**Figure 4:**
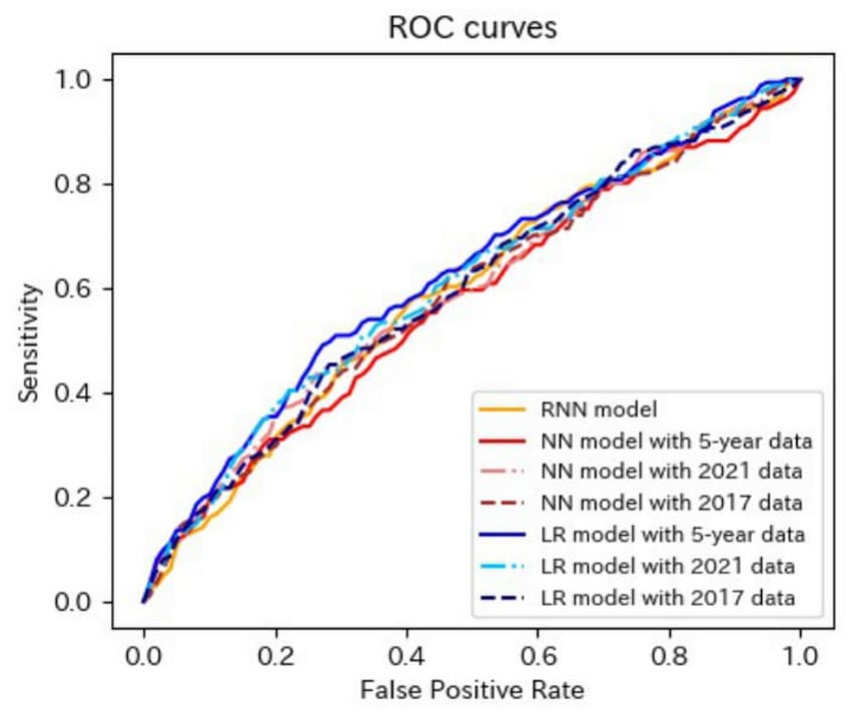
ROC curves of the models for the prediction of proteinuria in non-matched data analysis. ROC, receiver operating characteristics; RNN, recurrent neural network; NN, neural network; LR, logistic regression.

**Figure 5:**
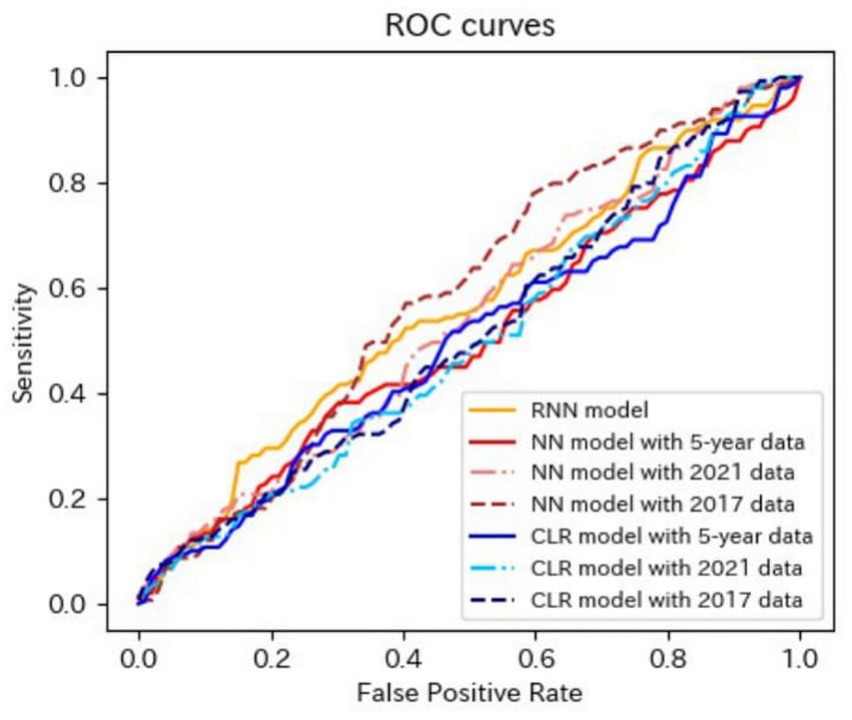
ROC curves of the models for the prediction of proteinuria in matched data analysis. ROC, receiver operating characteristics; RNN, recurrent neural network; NN, neural network; CLR, conditional logistic regression.

**Figure 6:**
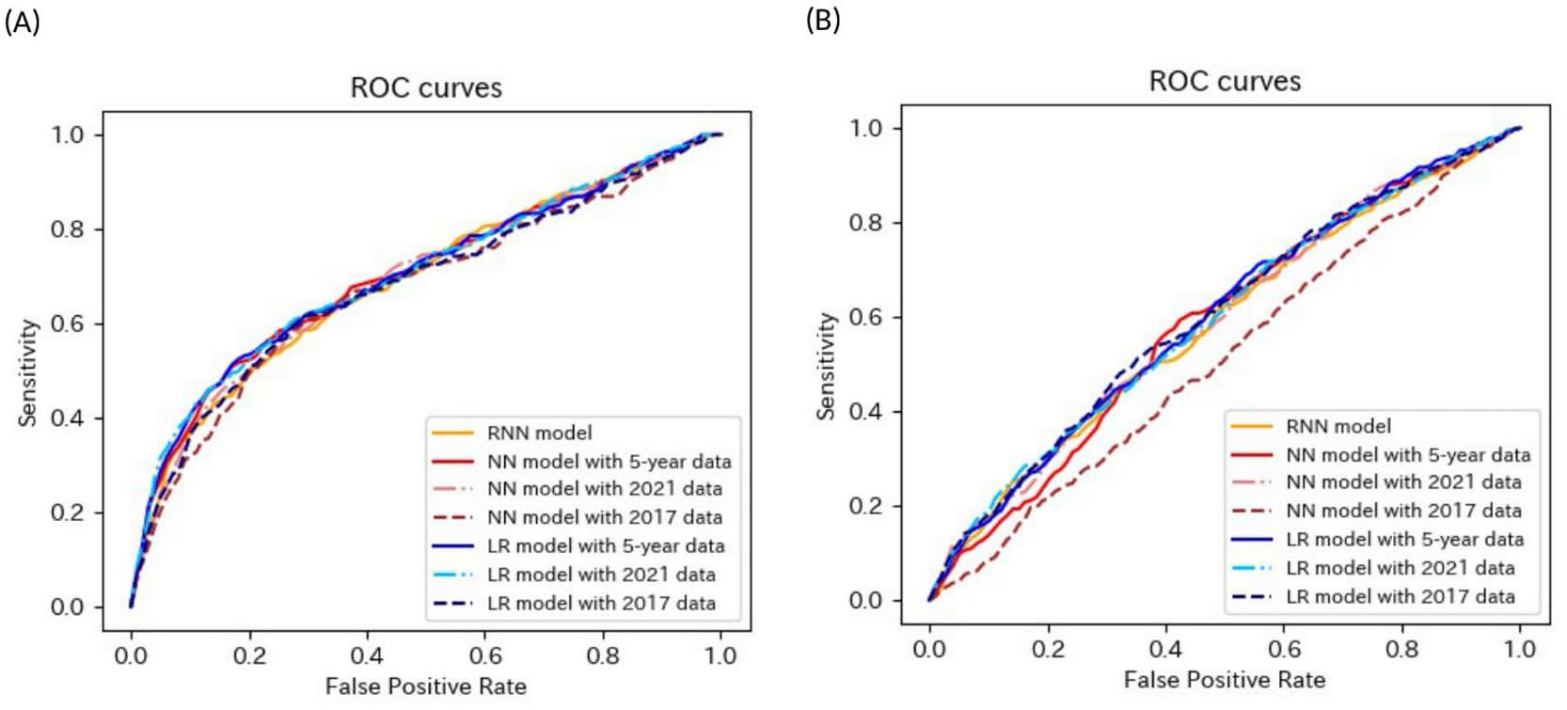
ROC curves of the models for the prediction of eGFR<60 mL/min/1.73 m^2^ or proteinuria in non-matched data analysis. (A) When all candidate variables are used as explanatory variables. (B) When eGFR was excluded from the explanatory variables. ROC, receiver operating characteristics; eGFR, estimated glomerular filtration rate; RNN, recurrent neural network; NN, neural network; LR, logistic regression.

**Figure 7:**
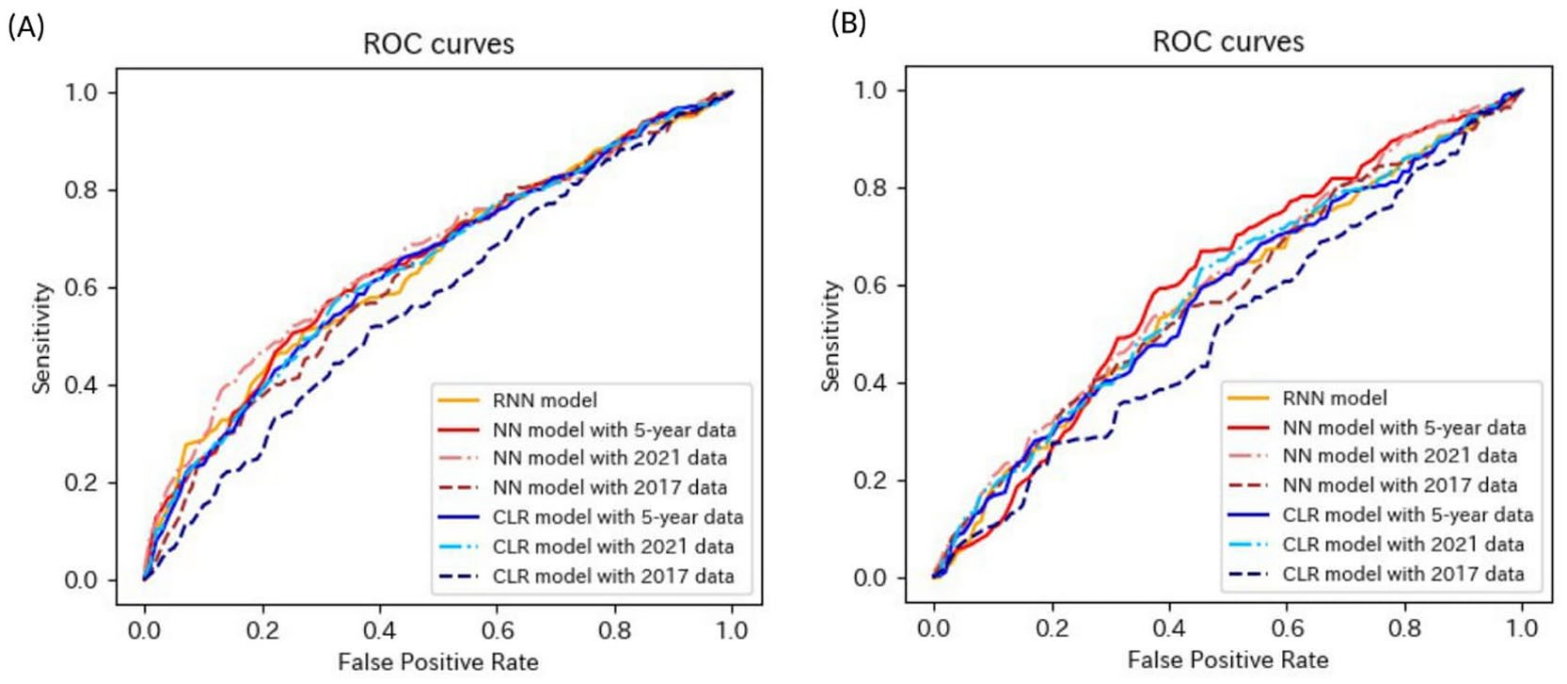
ROC curves of the models for the prediction of eGFR<60 mL/min/1.73 m^2^ or proteinuria in matched data analysis. (A) When all candidate variables are used as explanatory variables. (B) When eGFR was excluded from the explanatory variables. ROC, receiver operating characteristics; eGFR, estimated glomerular filtration rate; RNN, recurrent neural network; NN, neural network; CLR, conditional logistic regression.

Tables 4–6 present the predictive performance of the models. Figures 2–7 present the ROC curves of the models.

**Table 4:** Comparison between the predictive performance of models for the prediction of eGFR <60 mL/min/1.73 m^2^.

Non-matched data analysis					
Model	Explanatory variables	Accuracy	Sensitivity	Specificity	AUROC
RNN	All	0.839	0.934	0.837	0.941
	eGFR was excluded	0.659	0.653	0.659	0.703
NN with 5-year data	All	0.884	0.868	0.884	0.938
	eGFR was excluded	0.554	0.793	0.549	0.716
NN with 2021 data	All	0.856	0.901	0.855	0.935
	eGFR was excluded	0.766	0.554	0.771	0.722
NN with 2017 data	All	0.752	0.909	0.749	0.889
	eGFR was excluded	0.640	0.496	0.643	0.571
LR with 5-year data	All	0.826	0.942	0.824	0.941
	eGFR was excluded	0.519	0.860	0.512	0.732
LR with 2021 data	All	0.840	0.926	0.838	0.940
	eGFR was excluded	0.565	0.785	0.561	0.727
LR with 2017 data	All	0.743	0.934	0.739	0.890
	eGFR was excluded	0.559	0.793	0.554	0.729

Matched data analysis					
Model	Explanatory variables	Accuracy	Sensitivity	Specificity	AUROC
RNN	All	0.852	0.919	0.785	0.917
	eGFR was excluded	0.585	0.593	0.578	0.606
NN with 5-year data	All	0.844	0.904	0.785	0.908
	eGFR was excluded	0.626	0.615	0.637	0.638
NN with 2021 data	All	0.833	0.793	0.874	0.909
	eGFR was excluded	0.600	0.489	0.711	0.623
NN with 2017 data	All	0.759	0.770	0.748	0.810
	eGFR was excluded	0.585	0.459	0.711	0.596
CLR with 5-year data	All	0.811	0.919	0.704	0.877
	eGFR was excluded	0.556	0.378	0.733	0.507
CLR with 2021 data	All	0.819	0.844	0.793	0.887
	eGFR was excluded	0.548	0.326	0.770	0.507
CLR with 2017 data	All	0.767	0.807	0.726	0.803
	eGFR was excluded	0.570	0.385	0.756	0.563

Models with 5-year data are models with the summary statistics of data from 2017 to 2021.

AUROC, area under the receiver operating characteristic; RNN, recurrent neural network; eGFR, estimated glomerular filtration rate; NN, neural network; LR, logistic regression; CLR, conditional logistic regression.

**Table 5:** Comparison between the predictive performance of models for the prediction of proteinuria.

Non-matched data analysis					
Model	Explanatory variables	Accuracy	Sensitivity	Specificity	AUROC
RNN	All	0.588	0.584	0.588	0.590
NN with 5-year data	All	0.800	0.311	0.813	0.573
NN with 2021 data	All	0.736	0.435	0.744	0.594
NN with 2017 data	All	0.526	0.640	0.523	0.587
LR with 5-year data	All	0.725	0.491	0.731	0.621
LR with 2021 data	All	0.770	0.404	0.780	0.607
LR with 2017 data	All	0.714	0.453	0.721	0.595

Matched data analysis					
Model	Explanatory variables	Accuracy	Sensitivity	Specificity	AUROC
RNN	All	0.560	0.523	0.597	0.562
NN with 5-year data	All	0.540	0.356	0.725	0.505
NN with 2021 data	All	0.547	0.738	0.356	0.539
NN with 2017 data	All	0.594	0.779	0.409	0.583
CLR with 5-year data	All	0.523	0.517	0.530	0.499
CLR with 2021 data	All	0.527	0.980	0.074	0.498
CLR with 2017 data	All	0.537	0.973	0.101	0.512

Models with 5-year data are models with the summary statistics of data from 2017 to 2021.

AUROC, area under the receiver operating characteristic; RNN, recurrent neural network; NN, neural network; LR, logistic regression; CLR, conditional logistic regression.

**Table 6:** Comparison between the predictive performance of models for the prediction of eGFR <60 mL/min/1.73 m^2^ or proteinuria.

Non-matched data analysis					
Model	Explanatory variables	Accuracy	Sensitivity	Specificity	AUROC
RNN	All	0.779	0.509	0.792	0.690
	eGFR was excluded	0.642	0.480	0.649	0.582
NN with 5-year data	All	0.811	0.516	0.825	0.699
	eGFR was excluded	0.572	0.600	0.571	0.585
NN with 2021 data	All	0.827	0.465	0.844	0.690
	eGFR was excluded	0.686	0.444	0.698	0.589
NN with 2017 data	All	0.742	0.575	0.750	0.673
	eGFR was excluded	0.267	0.796	0.242	0.514
LR with 5-year data	All	0.811	0.524	0.825	0.700
	eGFR was excluded	0.463	0.709	0.451	0.596
LR with 2021 data	All	0.746	0.585	0.753	0.701
	eGFR was excluded	0.438	0.716	0.425	0.594
LR with 2017 data	All	0.708	0.615	0.713	0.680
	eGFR was excluded	0.621	0.538	0.625	0.600

Matched data analysis					
Model	Explanatory variables	Accuracy	Sensitivity	Specificity	AUROC
RNN	All	0.620	0.505	0.735	0.646
	eGFR was excluded	0.576	0.531	0.622	0.577
NN with 5-year data	All	0.629	0.575	0.684	0.651
	eGFR was excluded	0.607	0.593	0.622	0.603
NN with 2021 data	All	0.636	0.462	0.811	0.664
	eGFR was excluded	0.582	0.535	0.629	0.601
NN with 2017 data	All	0.604	0.553	0.655	0.629
	eGFR was excluded	0.565	0.345	0.785	0.577
CLR with 5-year data	All	0.613	0.607	0.618	0.640
	eGFR was excluded	0.571	0.593	0.549	0.574
CLR with 2021 data	All	0.624	0.567	0.680	0.642
	eGFR was excluded	0.589	0.633	0.545	0.588
CLR with 2017 data	All	0.567	0.520	0.615	0.572
	eGFR was excluded	0.538	0.276	0.800	0.511

Models with 5-year data are models with the summary statistics of data from 2017 to 2021.

AUROC, area under the receiver operating characteristic; RNN, recurrent neural network; eGFR, estimated glomerular filtration rate; NN, neural network; LR, logistic regression; CLR, conditional logistic regression.

### 3.1 Prediction of eGFR <60 mL/min/1.73 m^2^

The mean age of the participants with CKD was 50.1 years before matching, and 58.9% were men. The mean age of the participants without CKD was 44.5 years before matching, and 63.3% were men.

The accuracies, sensitivities, and specificities of all models were > 0.8 for predicting the onset of CKD in 1 year. The AUROCs of the models were > 0.9. However, the accuracy and specificity of the models were lower for predicting the onset of CKD within 5 years (<0.8). The models failed to attain AUROCs of 0.9, although the sensitivities were high (>0.9). The RNN and NN models with 5-year data and the NN model with 2021 data achieved AUROCs of >0.9 for matched data analysis; however, the AUROCs of all models were lower than those achieved for non-matched data analysis. All models exhibited accuracies of >0.8 for predicting the onset of CKD in 1 year. However, the sensitivity of the NN model with the 2021 data and the specificities of the models, except for this model, failed to reach 0.8. Both models exhibited lower sensitivity for predicting the onset of CKD within 5 years. The accuracy, sensitivity, specificity, and AUROC of the models, except for those of the CLR model with 2017 data in the matched data analysis, decreased when eGFR was not included as an explanatory variable. The NN model with the 2017 data exhibited a sensitivity of <0.5 in non-matched data analysis. The NN model with 2021 data, CLR model with 5-year data, CLR model with 2021 data, NN model with 2017 data, and CLR model with 2017 data exhibited a sensitivity of <0.5 in matched data analysis.

### 3.2 Prediction of proteinuria

The mean age of the participants with CKD was 44.3 years before matching, and 65.7% were men. The mean age of the participants without CKD was 44.7 years before matching, and 63.2% were men.

The AUROCs of all the proteinuria prediction models were lower than those of the models for the prediction of eGFR <60 mL/min/1.73 m^2^. The AUROCs of all models, except for those of the LR model with 5-year data and the LR model with 2021 data in the non-matched data analysis, were < 0.6. The sensitivity and specificity were also low. The sensitivities of the NN model with 5-year data, NN model with 2021 data, LR model with the 5-year data, LR model with 2021 data, LR model with 2017 data in the non-matched data analysis, and NN model with the 5-year data in the matched data analysis were < 0.5. The specificities of the NN model with 2021 data, the NN model with 2017 data, the CLR model with 2021 data, and the CLR model with 2017 data in the matched data analysis were < 0.5. The accuracy of the NN model with 5-year data in the matched data analysis was the highest (0.800).

These results indicated that the predictive performance of the models for proteinuria was worse than that of the models for eGFR <60 mL/min/1.73 m^2^.

### 3.3 Prediction of eGFR <60 mL/min/1.73 m^2^ or proteinuria

The mean age of the participants with CKD was 47.0 years before matching, and 62.2% were men. The mean age of the participants without CKD was 44.5 years before matching, and 63.3% were men.

Most models exhibited sensitivities of <0.6 and low AUROCs of approximately 0.65 in non-matched data analysis, even when all candidate variables were explanatory variables. The accuracies of the following models failed to reach 0.5 when eGFR was not included as an explanatory variable: the NN model with the 2017 data, LR model with the 5-year data, and LR model with the 2021 data in non-matched data analysis. The sensitivities of the following models failed to reach 0.5 when eGFR was not included as an explanatory variable: the RNN model, NN model with 2021 data in the non-matched data analysis, NN model with 2017 data, and CLR model with 2017 data in the matched data analysis. The specificities of the following models failed to reach 0.5 when eGFR was not included as an explanatory variable: the NN model with the 2017 data, LR model with the 5-year data, and LR model with the 2021 data in the non-matched data analysis.

These results indicate that the models for the prediction of eGFR <60 mL/min/1.73 m^2^ or proteinuria performed better than those for the prediction of proteinuria and worse than those for the prediction of eGFR <60 mL/min/1.73 m^2^.

## 4 Discussion

In this study, prediction models for the risk of incident CKD within 1 and 5 years were developed based on health examination data using RNN, NN, LR, and CLR. The predictive performances of the models were evaluated in terms of accuracy, sensitivity, specificity, and AUROC.

The predictive performance of the models using non-matched data was better than that of the models using age-and sex-matched data. This finding indicates that the distribution of factors varied between the CKD and non-CKD groups before matching and that the power of each factor improved significantly with an increase in the number of cases.

The RNN model performed similarly to the commonly used LR and NN models. The predictive performance of the RNN model was good even when age-and sex-matched data were analyzed. In particular, the AUROC of the RNN model was 0.917 in the prediction of eGFR <60 mL/min/1.73 m^2^. In the NN model, the weights of the variables were calculated between the input and hidden layers, between the hidden layers, and between the hidden and output layers. In the RNN model, in addition to the above weights, the weights between the hidden layer of a time step before and that of the next time step were calculated because the hidden layers were used repeatedly. This enables the RNN model to capture the changes in time-series data without using summary statistics such as the mean, slope of the regression line, and standard deviation. Thus, it is implied that the RNN model could capture the changes in health examination data and achieve a good predictive performance, similar to other models that require the calculation of summary statistics. Zhu et al. (20) used longitudinal electronic health records from patients with CKD. An RNN model was developed based on demographic characteristics, physical measurements, laboratory test results, and health behaviors to predict the risk of CKD progression from stages II/III to IV/IV. This model achieved an AUROC of 0.967 for predicting CKD progression within 1 year. These findings indicate that the RNN model and time-series data are highly effective tools for predicting future disease risk.

The predictive performance for predicting eGFR <60 mL/min/1.73 m^2^ was the best, followed by that for predicting eGFR <60 mL/min/1.73 m^2^ or proteinuria. The predictive performance for proteinuria was the worst. Thus, the predictive performance worsened when proteinuria was included as an outcome. The low sensitivity of dipstick tests used for the evaluation of proteinuria in health examinations was revealed by investigating the accuracy of the diagnosis of proteinuria using the dipstick test among Japanese workers (25). In addition, some individuals develop proteinuria without kidney injury (26). Therefore, many participants may have had false negatives on the dipstick test. Furthermore, as some participants developed proteinuria without kidney injury, it may be difficult to identify the differences between participants with kidney injury and those without kidney injury when developing proteinuria was the outcome.

The NN and LR models with 2021 (fifth-year) data demonstrated predictive performances similar to those of the RNN, NN, and LR models, which used longitudinal data for 5 years. In addition, when using all explanatory variables, the predictive performances of the NN and LR models with the 2017 (5 years before) data were similar to those of the NN and LR models in predicting the incidence of CKD within 1 year. In contrast, the predictive performances of all the models worsened significantly when eGFR was not included as an explanatory variable. These findings indicate that the patients had remarkably lowered eGFR values at least 5 years before the onset of CKD. Nelson et al. (27) developed risk prediction equations for incident CKD using the data from over 5 million individuals across 34 multinational cohorts and predicted the risk of a decline in estimated glomerular filtration rate (eGFR) to <60 mL/min/1.73 m^2^ within 5 years, based on demographic and clinical factors. The risk equations achieved a median C-statistic of 0.845, indicating good discrimination. Additionally, the findings indicated a significant association between low eGFR values and the incidence of eGFR falling to ≤60 mL/min/1.73 m^2^ in 5 years. These findings also suggest that the risk of developing CKD cannot be accurately predicted without eGFR. Miyakoshi et al. identified risk factors for CKD in Japan and reported that lowered eGFR (especially eGFR ≤70 mL/min/1.73 m^2^) was an overwhelmingly strong risk factor for predicting the incidence of CKD (28). In the present study, the predictive performances of models that predicted the risk of developing eGFR <60 mL/min/1.73 m^2^ within 5 years were inferior to those of models that made predictions within 1 year (for example, AUROCs of 5-year risk predicting models were < 0.9). Some people with high eGFR values may develop eGFR <60 mL/min/1.73 m^2^ in 5 years because of acutely decreasing eGFR, while some people with low eGFR values may maintain these low eGFR values without developing eGFR<60 mL/min/1.73 m^2^.

This study has three strengths. First, a large amount of health examination data that were accurately recorded every year were used. Second, the risk of developing CKD can be predicted well without the calculation of summary statistics using the RNN model, which has rarely been used. Third, the risk of CKD was predicted from the perspectives of both eGFR decline and proteinuria development, which revealed that predicting the risk of developing proteinuria was challenging.

However, this study had some limitations. First, whether eGFR <60 mL/min/1.73 m^2^ or the presence of proteinuria persisted over 3 months could not be confirmed in this study. Second, the information obtained through health examinations was limited. This study could not obtain information on some factors [e.g., family history of diseases (29, 30) and dietary intake (20)] suggested to be associated with the incidence of CKD in a previous study. Third, the proteinuria test conducted during the health examination was semi-quantitative, making it difficult to include proteinuria as an explanatory variable. Finally, the analysis period was short.

Machine learning models can predict the risk of CKD, and eGFR is a crucial factor in predicting the onset of CKD. However, predicting the incidence of proteinuria based solely on health examination data is difficult. The prediction models developed in this study may lead to awareness of risks and help individuals manage their health. However, further studies must be conducted to predict the decline in eGFR and increase in urine protein levels.

## Data Availability

The datasets presented in this article are not readily available because of patient privacy concerns. Interested researchers should contact the corresponding authors to inquire about access. The data have been made available with ethical approval. Requests to access the datasets should be directed to Kohei Akazawa, akzwkh0917@gmail.com.
